# A mass sacrifice of children and camelids at the Huanchaquito-Las Llamas site, Moche Valley, Peru

**DOI:** 10.1371/journal.pone.0211691

**Published:** 2019-03-06

**Authors:** Gabriel Prieto, John W. Verano, Nicolas Goepfert, Douglas Kennett, Jeffrey Quilter, Steven LeBlanc, Lars Fehren-Schmitz, Jannine Forst, Mellisa Lund, Brittany Dement, Elise Dufour, Olivier Tombret, Melina Calmon, Davette Gadison, Khrystyne Tschinkel

**Affiliations:** 1 Facultad de Ciencias Sociales, Universidad Nacional de Trujillo, Trujillo, Peru; 2 Department of Anthropology, Tulane University, New Orleans, LA, United Sates of America; 3 CNRS, UMR 8096 Archéologie des Amériques (CNRS-Paris1), Nanterre, France; 4 Department of Anthropology, Penn State College of the Liberal Arts, University Park, PA, United States of America; 5 Peabody Museum of Archaeology and Ethnology, Harvard University, Cambridge, MA, United States of America; 6 UCSC Paleogenomics Laboratories, Department of Anthropology, University of California Santa Cruz, Santa Cruz, CA, United States of America; 7 Comité Internacional de la Cruz Roja (CICR), Miraflores, Lima-Peru; 8 Sorbonne Universités, Muséum national d’histoire naturelle, CNRS, Paris, France; University of Florence, ITALY

## Abstract

Here we report the results of excavation and interdisciplinary study of the largest child and camelid sacrifice known from the New World. Stratigraphy, associated artifacts, and radiocarbon dating indicate that it was a single mass killing of more than 140 children and over 200 camelids directed by the Chimú state, c. AD 1450. Preliminary DNA analysis indicates that both boys and girls were chosen for sacrifice. Variability in forms of cranial modification (head shaping) and stable isotope analysis of carbon and nitrogen suggest that the children were a heterogeneous sample drawn from multiple regions and ethnic groups throughout the Chimú state. The Huanchaquito-Las Llamas mass sacrifice opens a new window on a previously unknown sacrificial ritual from fifteenth century northern coastal Peru. While the motivation for such a massive sacrifice is a subject for further research, there is archaeological evidence that it was associated with a climatic event (heavy rainfall and flooding) that could have impacted the economic, political and ideological stability of one of the most powerful states in the New World during the fifteenth century A.D.

## Introduction

Human and animal sacrifices were made by various societies in ancient world. In Prehispanic Peru, individuals were killed and placed in tombs to accompany important persons in the afterlife, buried as dedicatory offerings in monumental architecture, and sacrificed in various contexts as gifts to the gods [1–4]. Captives were taken in small-scale raiding and organized warfare, and killed in both formal rituals and impromptu reprisals [5–7]. Camelids also were sacrificed and deposited in burials as grave goods, buried as foundation or votive offerings and in other propitiatory contexts [8–12]. In recent years, human and animal sacrifice has become an active area of research for bioarchaeologists and zooarchaeologists working in the Andean region [13–20]. The results of recent excavations at the Huanchaquito-Las Llamas site (also known as “Gramalote A”) provide evidence of a previously unknown ritual involving a massive sacrifice of children and camelids by the Chimú State, c. AD 1450.

### The late intermediate period

The Late Intermediate Period (LIP, c. 900–1500 A.D.) was an unstable time in Peruvian North Coast prehistory marked by warfare and massacres as emergent polities fought for political, economic and religious control of the region. Archaeological evidence of conflict and subjugation includes a massacre at Punta Lobos in the Casma littoral of the Peruvian North Coast, where as many as 200 victims (children, adults and elderly) were executed during the military expansion of the Chimú state into the southern Casma region around AD 1350 [21]. Excavations at the Chimú capital of Chan Chan in the 1970s encountered the remains of hundreds of young women who were sacrificed to accompany their kings in royal burial platforms located in the palaces of Chan Chan [22–23]. Recent archaeological research at various archaeological sites north of the Casma and Moche valleys also has documented a growing number of human sacrifices dating between the 11^th^ and 15^th^ centuries AD in the Lambayeque region, at monumental sites and in isolated locations such as hilltops [16, 14, 15].

### The chimú state

**Fig 1:**
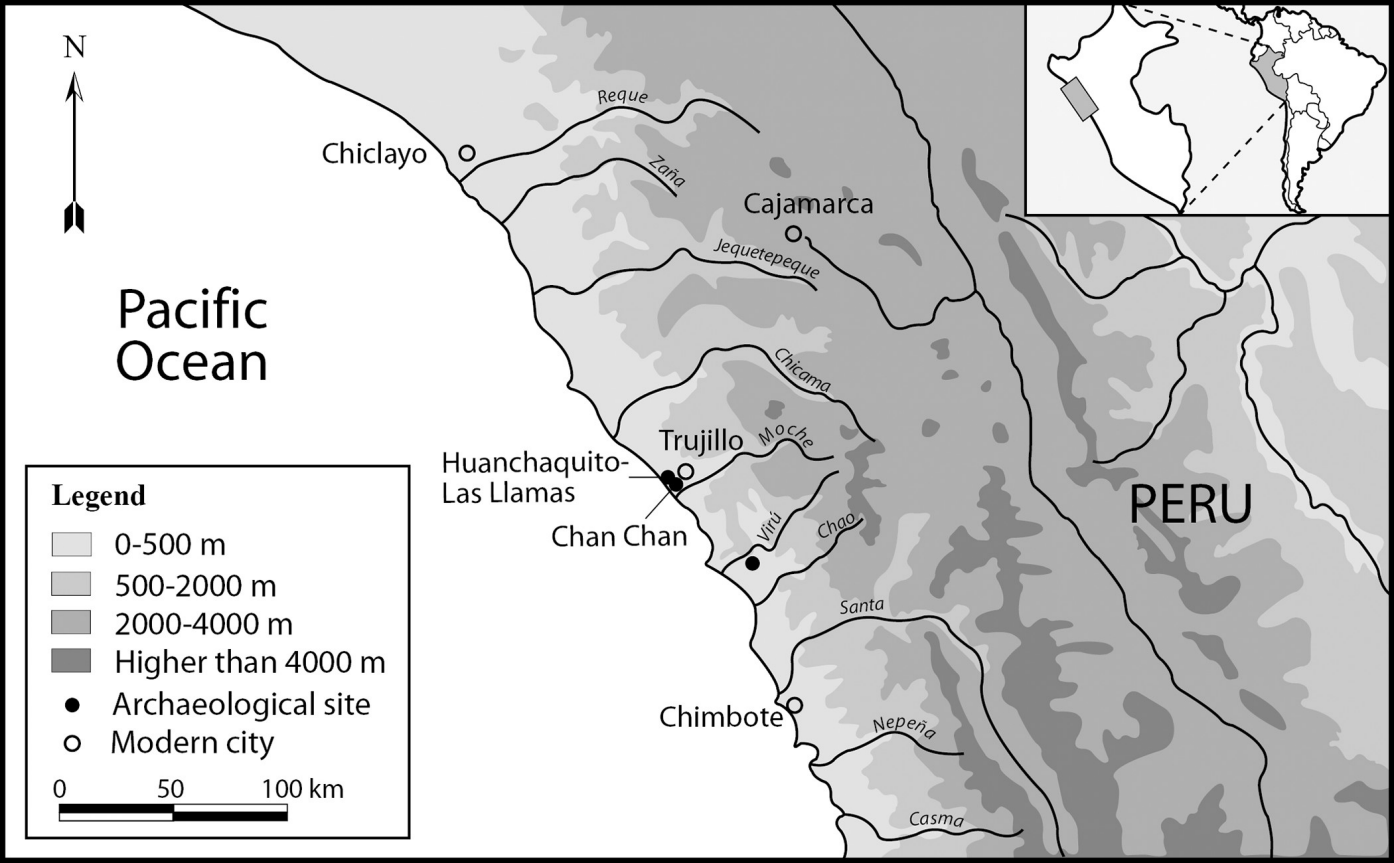
Map of the north coast of peru showing the extent of the chimú state and the geographic location of the huanchaquito-las llamas site.

The Chimú state flourished between the 11th and 15th centuries AD, dominating a broad expanse of the Peruvian coast. At its apogee, it controlled coastal valleys as far north as the present-day border of Peru and Ecuador and to the south as far as the present day Peruvian capital of Lima, encompassing more than 1000 kilometers of the Peruvian coastline (Fig 1). There is increasing evidence that Chimú territorial control extended into the neighboring highlands. The abundance of macaw and other tropical bird feathers in the ritual paraphernalia of the Chimú elite as well as the presence of toxic seeds from the same region (*Nectandra sp* and *Tevethia peruviana*), suggest that the Chimú exchange network reached as far eastward as the cloud forest and eastern slopes of the Andes [24]. Chimú hegemony was supported by intensive agriculture, with fields fed by a sophisticated web of hydraulic canals managed by an efficient bureaucracy. Crops and sumptuary goods were transported to well-organized storage facilities in cities and provincial administrative centers [24–28].Chan Chan is the name given today to the ancient capital of the Chimú state. It is located in the northern margin of the Moche valley, only a few miles away from the modern city of Trujillo. Chan Chan was one of the largest urban settlements of the Americas, and includes large palaces built by the successive kings, as well as administrative compounds, plazas, cemeteries, gardens, and temples linked by a network of internal roads [24]. Although today the surviving ruins of Chan Chan cover approximately 14 square kilometers, the city was once substantially larger; approximately six square kilometers of the site has been destroyed by modern agricultural and urban expansion. Additional shrines, satellite settlements and other structures were located on the outskirts of the city as well [23]. The strategic location of Chan Chan between the Pacific Ocean, wetlands, agricultural fields, desert and mountains, suggest that its builders envisioned it as dynamically interacting with the landscape, integrating these elements into the rituals performed within and around the city’s walls [28].

### Child sacrifice

Infant and child sacrifice is claimed to have been practiced by many ancient societies, although archaeological evidence of intentional killing is often lacking, making these interpretations subject to debate. Old World archaeological evidence of child sacrifice is less than convincing in many cases [29–32], and in North America and Mesoamerica the evidence is frequently ambiguous as well [33], although there are a growing number of convincing examples from the Maya area [34–35]. The most well-documented archaeological evidence of child sacrifice in the New World is known from Offering 48 at the Templo Mayor in the Mexica city of Tenochtitlan [36].

The motivations for human sacrifice and the choice of sacrificial victims varied among ancient societies, but anthropologists have noted that children’s bodies frequently are considered as hybrid entities [37], and thus may have been viewed as particularly appropriate as messengers or gifts to the gods [38].

In Andean South America, sacrifices of children are known to have been made by the Inca and by some societies that came before them. Although no archaeological evidence has been found to confirm ethnohistoric accounts claiming that large numbers of children were sacrificed by the Inca on particular occasions, such as the death or coronation of an Inca ruler [39], a small number of child sacrifices have been recovered on high mountaintops in recent decades in excavations conducted by international research teams [38, 40]. Examples of Inca child sacrifices also have been reported from the Cuzco region and north coast of Peru [13]. These discoveries have provided an opportunity to directly compare archaeological evidence to ethnohistoric accounts of the Inca practice of “Capacocha” mountain sacrifices, and some innovative analytical methods have been employed to examine questions such as the geographic origin and life histories of the children and the source of offerings buried with them [41–43].

### Child sacrifice in northern coastal peru

Until the Huanchaquito-Las Llamas discovery there was very little archaeological evidence of human sacrifices on the north coast of Peru that included only [15]. Ethnohistoric sources likewise are limited to an account by the Spanish Friar Antonio de la Calancha, who claimed that child sacrifices were made by the Chimú in the Jequetepeque river valley during lunar eclipses, along with offerings of fruits, maize beer and colored cottons. Calancha also told the story of a local healer named “Mollep” near the Jequetepeque valley who sacrificed children to sacred places or “guacas” [44].

Archaeological discoveries of retainer and dedicatory burials and sacrificed captives have been made at multiple sites on the north coast [2, 6, 45], as well as sacrificial offerings that include a mix of children, adolescents, and adults [15], but until recently only one possible example a sacrifice containing only children and camelids was known. In 1969, excavations in the seaside town of Huanchaco by archaeologist Christopher Donnan encountered the remains of seventeen children and twenty camelids buried together in simple pits without funerary offerings [46]. Although osteological analysis was not done to determine possible cause of death, on the basis of their archaeological context, demographic profile, and atypical burial pattern, Donnan concluded that the burials probably were sacrificial offerings. A radiocarbon date placed the event at circa AD 1400 (UCLA-1879), during the Chimú domination of the North Coast [46]. No other reports of child sacrifices in the region were made until the discovery at Huanchaquito-Las Llamas, leaving this early find as an intriguing, but isolated case.

### Camelid sacrifice in northern coastal peru

Camelids were the principal animals used for sacrifices in the Central Andean region during Prehispanic times. Although some ritual deposits of camelids are known from as early as the Late Preceramic (prior to 1800 BC) at the Temple of Crossed Hands at Kotosh [47], the sacrifice of camelids dramatically increased during the Early Intermediate Period (c. 100–600 AD), particularly in the Moche culture of northern Peru [8–10]. The most common pattern is the inclusion of whole camelids or body parts (preferentially skull and leg extremities) as funerary offerings. In tombs, they played both alimentary and symbolic functions. Mass sacrifices of camelids are rare; usually restricted to less than ten animals. However, a few examples of mass burials are known. At the site of Cahuachi in the Nazca valley in the Southern Coast of Peru, 64 whole camelids were discovered in a single context [48] and at least 88 camelid skulls were found in a Moche context at the site of San Jose de Moro, Jequetepeque Valley, on the North Coast of Peru [9].

During the Chimú occupation of the Moche Valley, complete camelids were deposited alone or together with humans in tombs and in storage facilities at the Huaca de la Luna and at Chan Chan [49, 22]. However, the early discovery by Donnan and Foote in the late 1960’s and the present case described here suggest that Huanchaco served as a particular focus of child and camelid sacrificial offerings.

## Materials and methods

The archaeological materials described in this paper (specimen inventory numbers for human bones: E01 to E140, camelids: CA01 to CA200 and artifacts: Ce01, Ce02, Ot01 and Ot02) are currently housed at the deposits of the Chan Chan Museum and Huaca El Dragon storage facilities, both managed by the Ministry of Culture in Trujillo (Peru). The permits to conduct archaeological excavations at Huanchaquito Las Llamas and to export archaeological materials to conduct specialized analysis were granted by the Peruvian Ministry of Culture (RD-022-DGPC-VMPCIC/MC, June 13^th^, 2011; RD-009-2012-VMPCIC/MC, February 24^th^, 2012; RD-146-2014-DGPA-VMPCIC/MC, April 1^st^, 2014; RD-111-2014-VMPCIC/MC, October 22^nd^, 2014 and RD-092 2016-DGPA-VMPCIC/MC. March 14^th^, 2016). The archaeological specimens are publicly deposited at the Site Museum of Chan Chan and the Storage Facility located in the Huaca del Dragon Archaeological Site both managed by the Ministry of Culture in Trujillo (Peru) and accessible to the scientific team members and to any authorized researcher.

### The huanchaquito-las llamas sacrificial site

**Fig 2:**
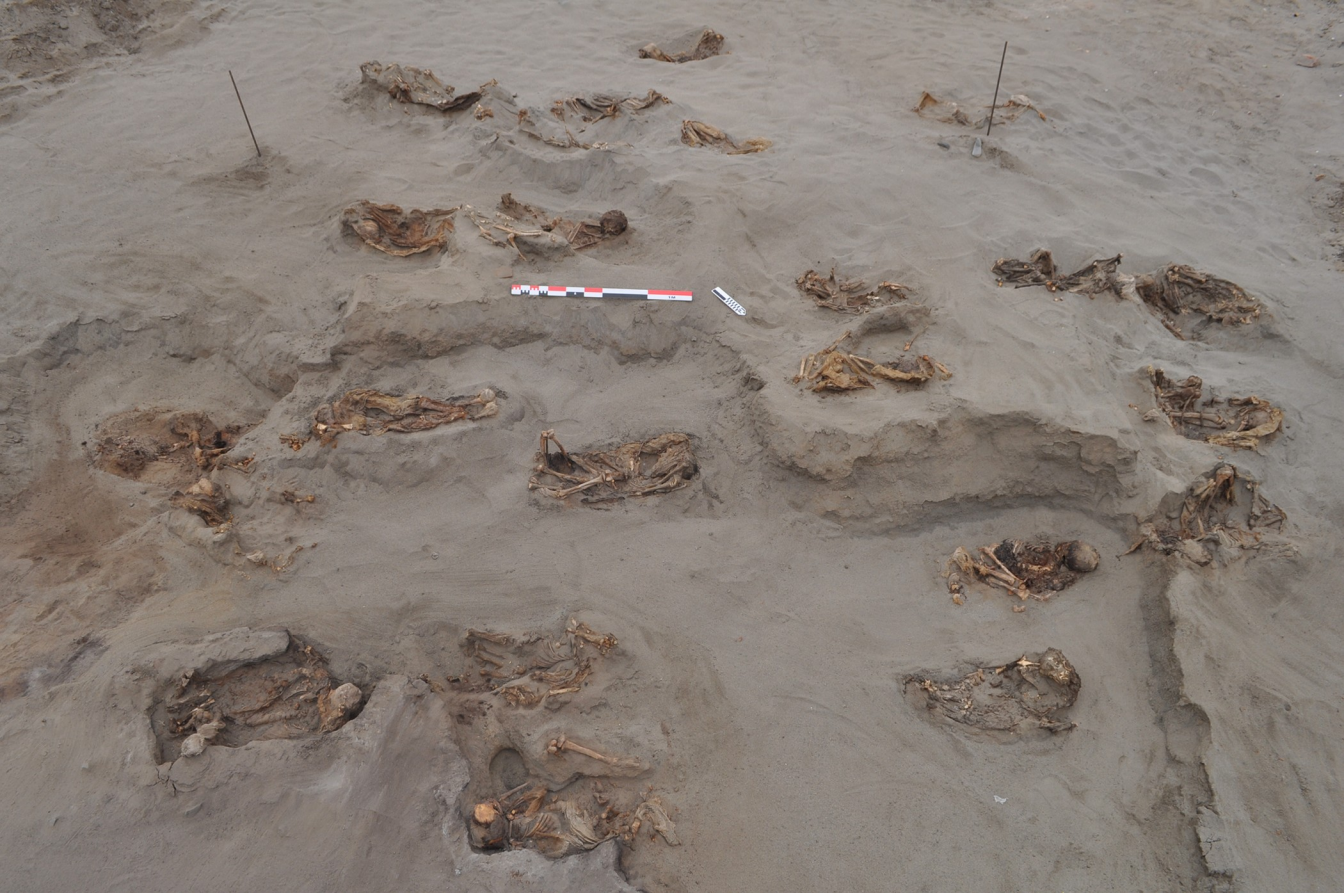
Child and camelid skeletons buried in windblown sand at the HLL site. Note that all the burials were excavated to relatively the same depth.

The archaeological site of Huanchaquito-Las Llamas (hereafter abbreviated as HLL) is located in the Department of La Libertad, Province of Trujillo, District of Huanchaco, in northern coastal Peru (UTM coordinates: 9’104,494.00 N– 708.824.00 E) (Figs 1 and 2). The site, located 350 meters from the shoreline, is a deposit of windblown beach sand covering the lower flank of marine terrace that reaches a height of approximately eleven meters above sea level and is located 2 miles north of the city of Chan Chan. The site is delimited on the south by modern construction and to the north by an area used as a disposal area for construction debris and refuse. In the late 1990s, the western portion of the site was cut through by heavy machinery during construction of a road. It can be assumed that a substantial, but unknown quantity of cultural materials buried along the margins of the site have been lost because of construction activity.

In 2011, in response to reports from local residents that human and camelids bones were eroding out of the roadside dunes, Prieto requested permission from Peru’s Ministry of Culture to conduct an emergency excavation to recover materials in danger of destruction through erosion and continued construction activities [20]. Excavations revealed no evidence of architecture or ancient habitation refuse, but encountered a concentration of burial pits containing the remains of 43 children and 74 camelids that based on radiocarbon determinations dated to the Late Intermediate Period (c. AD 1450) Chimú occupation of the north coast (Table 1).

**Table 1:** Available C14 dates.

Huanchaquito Las Llamas	Curve ShCal04				Unmodelled (BC/AD)					
					1 sigma			2 sigma		
Lab number	Field Number	Material sample	BP	±	From	To	%	from	to	%
R_Date 120948	E2	Collagen	630	15	1325	1398	68.2	1319	1404	95.4
R_Date 120949	E4	Collagen	475	15	1445	1455	68.2	1434	1464	95.4
R_Date 120950	E6	Collagen	525	15	1425	1442	68.2	1416	1448	95.4
R_Date 6182 (PSU number)	CA 25	excrement	865	25	1201	1233	43%	1180	1271	95.4
R_Date 6183 (PSU number)	E40	organic material	485	20	1438	1455	68.2	1425	1464	95.4
R_Date 6184 (PSU number)	CA 52	Textile	505	20	1430	1450	68.2	1419	1455	95.4
R_Date 6442 (PSU number)	MF 15	Sedge rope	520	20	1426	1445	68.2	1417	1450	95.4
R_Date Beta-396743	CA 102	Sedge rope	540	30	1413	1440	68.2	1400	1450	95.4
R_Date Beta-396744	CA 107	Sedge rope	520	30	1431	1457	68.2	1413	1482	95.4
R_Date Beta-396745	CA 69	Sedge rope	450	30	1449	1496	68.2	1436	1622	95.4
R_Date Beta-396746	E92	Sedge rope	400	30	1449	1496	68.2	1436	1622	95.4
R_Date 6543 (PSU number)	CA 80	Sedge rope	505	15	1433	1448	68.2	1425	1454	95.4
R-Date 6544 (PSU number)	CA 81	Sedge rope	485	15	1441	1454	68.2	1433	1459	95.4
R_Date 6545 (PSU number)	CA 85	Sedge rope	540	15	1418	1436	68.2	1410	1443	95.4
R-_Date 6546 (PSU number)	CA 96	Sedge rope	555	15	1410	1429	68.2	1404	1437	95.4
R_Date 1269 (PSU number)	CA 187	Sedge rope	565	30	1403	1430	68.2	1391	1447	95.4
R_Date 1270 (PSU number)	CA 159	Sedge rope	620	30	1389	1406	68.2	1378	1424	95.4
R_Date 1271 (PSU number)	CA 199	Sedge rope	625	30	1386	1403	68.2	1378	1419	95.4
R_Date 1272 (PSU number)	E133-2	Human hair	985	20	1101	1149	68.2	1034	1154	95.4
R_Date 1608 (PSU number)	E24	Collagen	515	15	1430	1445	68.2	1420	1450	95.4
R_Date 1272 (PSU number)	E31	Collagen	625	15	1326	1401	68.2	1320	1406	95.4

Archaeological context indicated that the children and camelids buried at HLL were sacrificial victims. Unusual burial positions and a lack of any associated grave goods suggested that this was not a typical Chimú burial ground. In addition, analysis of ten of the children’s skeletons found that half of them had cuts transecting the sternum [20]. This, in conjunction with field observations of displaced ribs suggested that the chest had been cut open, perhaps to extract the heart. Similar cut marks were found on the ribs and sterna of many of the camelid skeletons as well [19].

**Fig 3:**
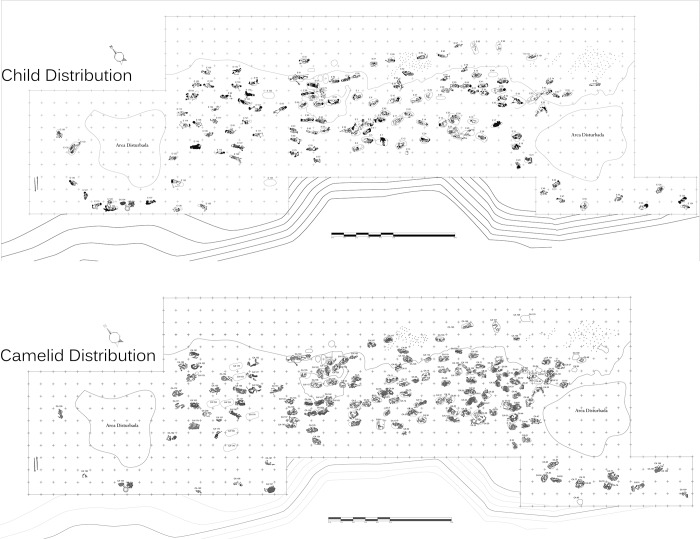
Maps of the distribution of children (n = 137 + 3 adults) and camelids (n = 200).

In 2014 and 2016 funding was obtained to do further excavation at the site and to perform a detailed analysis of the human and camelids skeletons recovered in 2011, along with those excavated in 2014 and 2016. The original excavation area was expanded by six additional units (Fig 3). Excavations in 2014 and 2016 effectively tripled the number of sacrificial victims, resulting in a final count of 140 individuals (137 children and 3 adults) and 200 camelids. The three adult skeletons found in 2014 at HLL are distinctive, not only for their age at death but also for their manner of death and the burial position of the two females (E59, 20–30 years old; E69, 18 years old). Unlike the HLL children they do not have cut sterna or spread ribs, indicating that their chests were not opened. One of the females appears to have died from a blow to the back of the head; the other suffered blunt force trauma to the face, but no cause of death was identifiable from the skeleton. The adult male (E91, 30–40 years old) has fractures of multiple ribs, although it is not clear whether they are perimortem or postmortem fractures produced by the weight of a pile of rocks that was placed over the body when it was buried; no cause of death could be identified. Both females were buried crouched on their knees, face down, while the male was buried extended on his back. All three were placed in close proximity to one another and to sacrificed children at the northwest margin of the site, and thus they appear to have been associated with the sacrificial event.

The total number of young individuals is higher if we count incomplete remains recovered from areas disturbed by recent human activity at the site.

Complete excavation of the site indicates that the children and camelids were buried in an area of approximately 700 square meters (50 meters N-S axis and 14 meters E-W axis). The southern, northern and western edges of the site have been severely impacted by modern construction, destroying the human and animal contexts (Fig 2). Nevertheless, our excavations confirmed that the bulk of the sacrificial victims (both human and animal) were concentrated in the central portion of the site. Although a first impression would suggest a continuous distribution of the sacrificial victims, there are four identifiable clusters of human and animals, not only two as was originally suggested based on excavations done during the initial field season [20].

**Fig 4:**
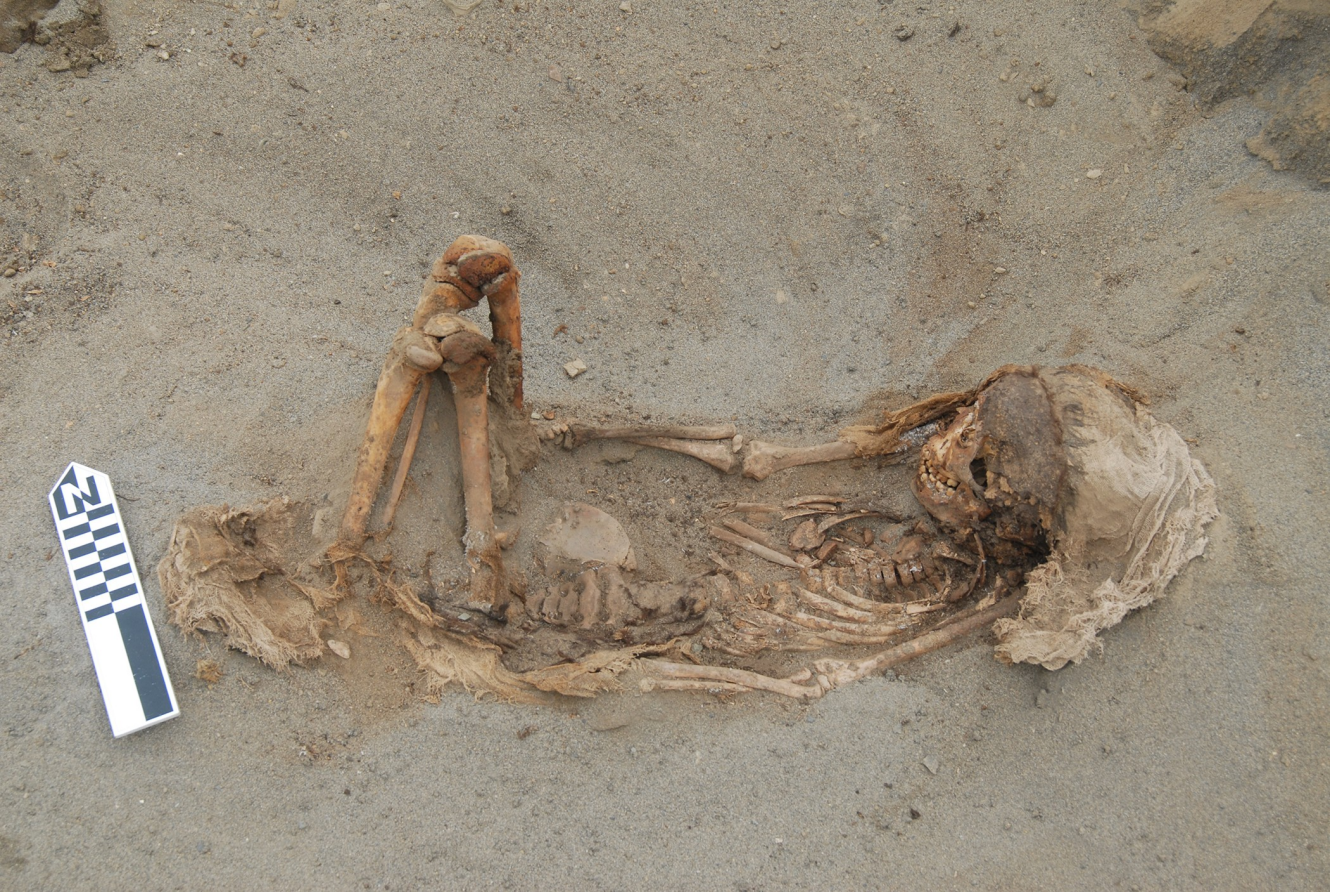
A typical child burial laying on its back with the legs flexed. Note the cloth on the head. The body was placed facing northwest, toward the ocean.

**Fig 5:**
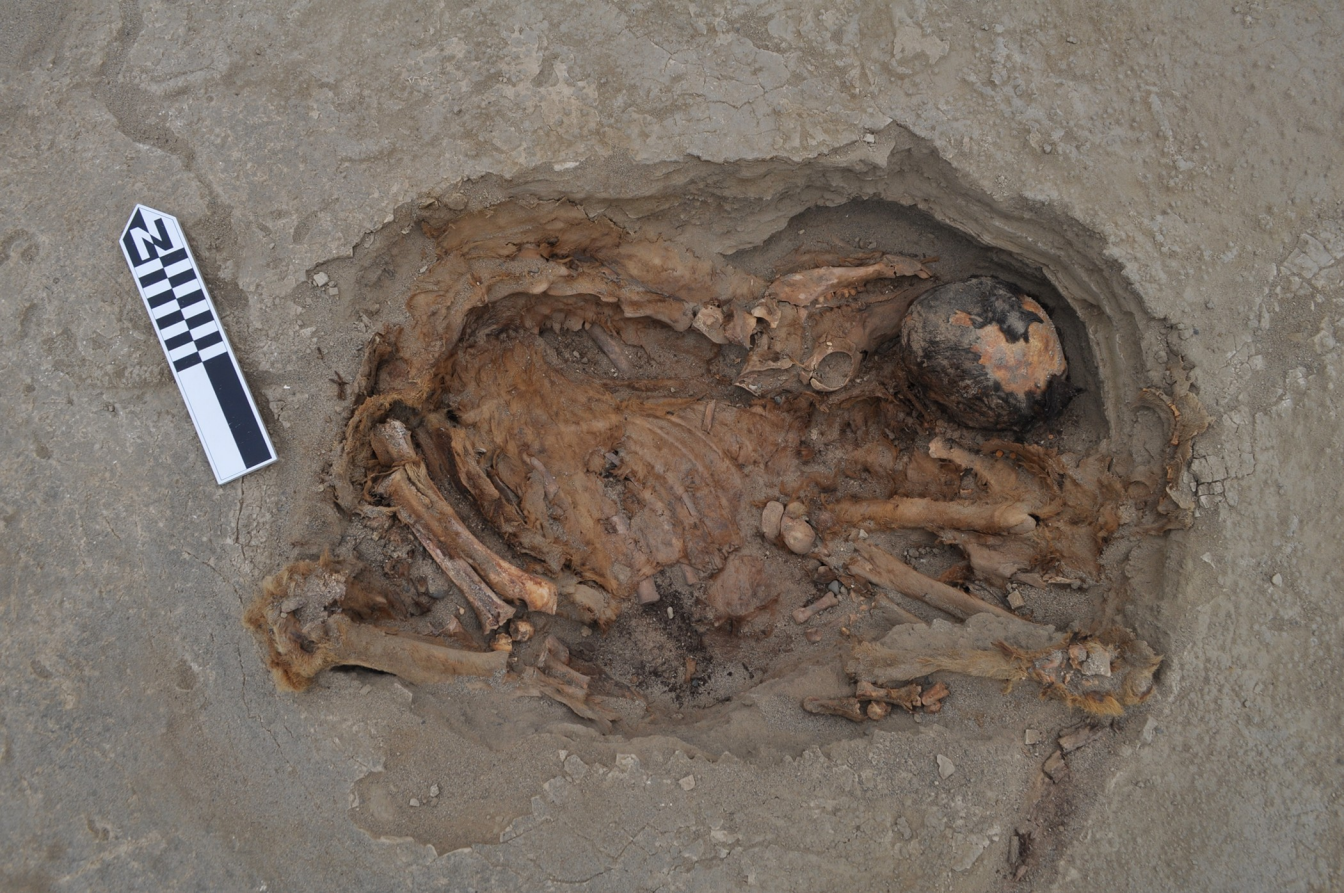
A light-brown camelid laying on top of a human body. Like most of the camelids, the hair is very well preserved.

Detailed study of the body positions of the children as well as age at death and form of cranial deformation do not reveal identifiable patterns that distinguish the four clusters from one another. A similar observation was made for the camelids, suggesting some other explanation for the four distinct clusters of burials. Some consistent patterns in burial position and differential treatment of children and camelids were noted, however. Preliminary analysis suggests that humans and camelids were buried following a strict order in which most of the children faced to the northwest (the sea) and camelids toward the mountains [20]. The children were wrapped in simple plain weave cotton shrouds and were buried in one of three body positions: resting on their backs with lower extremities flexed, flexed resting on one side and extended on the back. Children often were buried in groups of three and placed by increasing age from youngest to oldest. Some children received special treatment before being sacrificed. Some had their faces painted with a red cinnabar-based pigment, and others (primarily older children) wore distinctive cotton headdresses (Fig 4). Camelids were carefully accommodated next to or on top of the human bodies. In many cases camelids of contrasting colors (brown and beige) were buried together, placed in different orientations (Fig 5).

**Fig 6:**
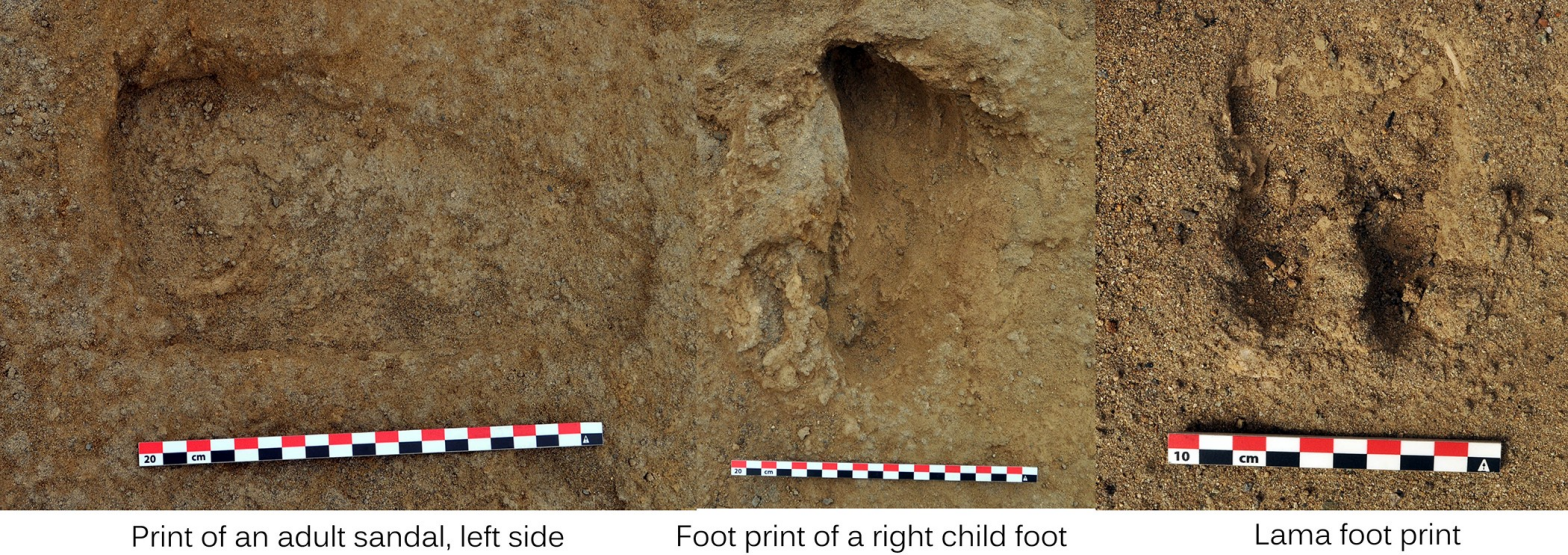
Examples of human (bare foot of a child and sandal print of an adult) and camelid footprints in wet mud.

Along the east side of the four identified burial clusters lay a dried mud surface. It appears that this deposit of mud originally covered the entire site, but the excavation of burial pits for the sacrificial victims and the apparent heavy transit during the sacrificial event may have destroyed portions of it. This was suggested by the presence of numerous fragments of dried mud in areas where the surface was not intact. Only a few children and camelids were buried on the eastern portion of the site, and in this area the mud was well preserved. Well-preserved human, camelid and dog foot prints were made on this mud surface while it was still wet (Fig 6). Some of the human footprints are identifiable as impressions of adult sandals while smaller footprints were made by children who walked barefoot (no sandals were found with the sacrificial victims). The size and shape of the camelid footprints match well with the estimated size of a young camelid hoof, suggesting that the animals who marked their transit through the site were sacrificial victims. The orientation of the footprints indicates that at least two large groups of children and camelids came from the northern and southern sides of the site to meet at the center of the sacrificial area. From there, the sacrifice presumably was conducted and the human and animal bodies placed in burial pits excavated through the mud layer. On a few cases, some children and camelids were left on the surface of the mud layer which was still in the process drying. These data suggest that the sacrificial event took place shortly after a heavy rain and flood event that covered the entire surface of the sand dune with a layer of mud, clay and gravel.

An aerial photograph taken in 1942 shows the presence of at least two dry erosional channels that extend from the top of the bluff to the area where the children and camelids were sacrificed. Preliminary analysis of the soil sediments found directly over the clean sand of the site suggests that the gravel and type of clay deposited are identical to the ones found on top of the bluff. Subsequent excavations identified water courses on the northern and southern limits of the site, confirming that the dry erosional channels observed in the 1942 aerial photograph were active during the climatic event of the fifteen century AD.

### Associated ceramics and wood implements

Although no offerings were found directly associated with the child and camelids deposits, a pair of ceramic jars and wooden paddles were found buried at the northern margin of the sacrificial site, accompanied by a single camelid. One jar was a black reduction-fired vessel decorated on its neck with a human face and two *Spondylus* shells. The jar appears to have been broken intentionally prior to burial, as it was found in numerous small pieces. The second jar, an undecorated oxidation-fired vessel was found next to the first. It was complete, with a sedge rope tied around its neck. The two wooden paddles were found near the ceramic vessels. Their function has been identified through Chimú art and through the contexts in which they have been found at other Chimú sites. A Chimú wooden architectural model depicting a funerary procession includes a depiction of individuals preparing a beverage on large ceramic vessels using wooden paddles, and at a contemporaneous site located in the northern Jequetepeque Valley, a Chimú brewery where ceramic vessels were carefully buried with wooden paddles on top of them was found by Prieto [50]. The ceramic vessels and wooden paddles found at HLL thus appear to be associated with the production and perhaps consumption of maize beer or *chicha* as part of the sacrificial ritual, after which the vessels and paddles were buried on site.

### Contemporaneity of the sacrificial event

One of the objectives of the excavations was to determine whether this massive concentration of sacrificed children and camelids represents a single event or a series of smaller events. Stratigraphic analysis indicates that all the humans and animals were buried in the same layer of clean sand. Almost all bodies were buried at the same depth and in close proximity to one another, and no examples were found of burial pits that cut into others. Only two bodies located on the northern sector of the site were found at a significantly greater depth. The discovery of human and camelid foot prints made in wet mud suggest that the victims circulated near the area where they were finally buried. These data, along with our observations on burial position and spatial clustering suggest that a) the children and the camelids were sacrificed at this location (rather than their bodies being brought from elsewhere) and that b) the final disposition of the human and animal bodies followed a consistent sacrificial program planned and organized, perhaps, by Chimú priests and officials.

### Dating the event

Twenty AMS radiocarbon determinations were made by two independent laboratories. The samples were drawn from different sectors of the excavations, and all are based on short-lived plant remains (sedge ropes associated with the camelids and cotton threads from children’s burial shrouds). Four C14 dates out of 21 are slightly earlier than the others, and they may have been the result of using different materials (for example human hair, collagen or camelid excrement) or simply standard errors in a large sample (Table 1). All in all, the results cluster around CAL AD 1400–1450 (Table 1). Using one or two sigma calibrations, the results indicate that the sacrificial event can be dated relatively precisely to this range of dates, placing it in the late Chimú period. Stratigraphic evidence suggests that the sacrifice was made following a heavy rain/flood event that deposited a layer of mud on top of the clean sand in which the children and camelids were buried. The mud appears to have been deposited as sheet wash during a major rainfall event (or series of events), and is probably associated with the El Niño-Southern Oscillation (ENSO) phenomenon or a similar climate alteration (“El Niño Costero” for instance) that periodically brings coastal flooding and elevated sea temperatures that disrupt the marine food chain in northern and central Peru. It is possible that the sacrifices were made in response to the heavy rains, as burial pits were dug through the mud layer and in a few cases some children and camelids were left on top of the wet mud.

### Osteological analysis of the sacrificed children

In the field laboratory a detailed dental, skeletal, and photographic record was produced for each individual. Age at death was estimated based on dental calcification and eruption, long bone length and epiphyseal fusion. The three adults were aged based on epiphyseal fusion and pubic symphysis morphology [51–52].

Skeletal and dental pathology, including developmental defects, infectious disease, trauma, and oral pathology was recorded by visual examination following standard guidelines [51–53]. The presence, absence, and form of cranial modification was also recorded. Finally, observations were made on perimortem injury and probable cause of death. With the exception of three adult burials (two females and one male), all the human skeletal remains were of children, ranging in age from approximately five to fourteen years, with the majority falling in the range of eight to twelve years of age.

Laboratory examination suggests (to the degree possible from skeletal and dental observations) that the children were in good health, with low frequencies and only mild expression of nutritional stress indicators commonly used as measures of childhood health [54–55]. Frequencies of porotic hyperostosis (14.1%), cribra orbitalia (16.5%), enamel hypoplasias (less than 10%) and periosteal reactions on long bones(10.6%) are all low compared to other skeletal samples from LIP and LH coastal Peruvian sites [56–58]. Dental caries and periapical abscesses were observed on some deciduous teeth, but no individuals showed notable oral pathology. These low frequencies do not suggest that marginalized or low social status children were preferentially selected for sacrifice at Huanchaquito, as appears to have been the case for some other north coast Peruvian sacrifices [15].

### aDNA preliminary analysis

Short hair and remains of loincloths worn by some individuals are suggestive of male sex, but skeletal morphology cannot distinguish males and females at this young age. However, preliminary analysis of dental samples using gonosomalDNA markers indicates that both boys and girls are present in the sample (Table 2). In a preliminary study, sex chromosomal markers were successfully analyzed for 28 individuals using a multiplex quantitative PCR (qPCR) assay amplifying three short intergenic sequences on both gonosomes: two y-chromosomal (44 and 47 bp), one x-chromosomal (44 bp) target [59]. For 20 of those individuals the presence of both X- and Y-chromosomal markers could be determined, while for eight individuals the analyses only revealed the presence of X-chromosomal markers in several replications, suggesting that these individuals were female. Because of the degraded nature of the DNA isolated from the specimens, alleleic dropout must be considered as a potential explanation for this lack of y-chromosomal signals from these eight individuals, but the consistent results found in at least four replications for each sample strongly support the accuracy of the results. We further confirmed the qPCR based genetic sex determinations for 7 individuals by exploring the ratio of X- to Y-chromosomal reads [60] from low coverage shotgun sequencing data. We build double-stranded, partially UDG treated sequencing libraries [61] from the DNA extracts, and sequenced those for ~300,000 reads on a Illumina MiSeq Next Generation Sequencer (NGS) (see Table 3– NGS sequencing statistics). After demultiplexing, resulting sequencing reads were processed using the in-house computational pipeline developed for aDNA described in (Fehren-Schmitz et al. [62], which includes the assessment of DNA damage patterns and mitochondrial contamination rations [63–64]. We confirmed the Native American ancestry of the individuals by determining their mitochondrial haplogroups (Tables 2 and 3) using a multiplex single-base extension PCR assay [65]. Further, genome wide sequencing analyses are currently in progress in order to explore the population genetic affinities of the sacrificed individuals.

**Table 2:** Genetic sexing results for the ancient huanchaquito las llamas samples and mitochondrial haplogroups.

Sample Name	Biological Sex	TriXY qPCR	NGS	mtHaplogroup
**LLS_E3**	Female	0Y, 1X	XX	D1
**LLS_E07**	Female	0Y, 1X	XX	A2
**LLS_E10**	Male	2Y,0X	n.d.	D1
**LLS_E17**	Possible male	1Y, 1X	n.d.	D1
**LLS_E19**	Male	2Y, 1X	n.d.	A2
**LLS_E21**	Male	2Y, 1X	XY	D1
**LLS_E24**	Male	2Y, 1X	XY	D1
**LLS_E28**	Female	0Y, 1X	n.d.	C1b
**LLS_E31**	Possible male	1Y, 1X	Not assigned	C1d1
**LLS_E34**	Possible male	1Y, 1X	XY	D4h3
**LLS_E37**	Possible male	1Y, 1X	n.d.	C1b
**LLS_E40**	Possible female	0Y, 1X	n.d.	B2
**LLS_E43**	Possible male	1Y, 1X	n.d.	B2
**LLS_E48**	Possible male	1Y, 1X	n.d.	A2
**LLS_E50**	Male	2Y, 1X	n.d.	C1b
**LLS_E52**	Female	0Y, 1X	n.d.	A2
**LLS_E57**	Possible Male	1Y, 1X	n.d.	n.d.
**LLS_E59**	Possible male	1Y,1X	n.d.	C1d1
**LLS_E63**	Male	2Y, 1X	XY	D1
**LLS_E66**	Male	2Y, 0X	n.d.	D1
**LLS_E68**	Possible male	1Y, 1X	n.d.	n.d.
**LLS_E76**	Female	0Y, 1X	n.d.	A2
**LLS_E79**	Female	0Y, 1X	n.d.	B2
**LLS_E83**	Possible male	1Y, 1X	Not assigned	D1
**LLS_E84**	Possible male	1Y, 1X	n.d.	n.d.
**LLS_E89**	Possible male	1Y, 1X	n.d.	n.d.
**LLS_E90**	Female	0Y, 1X	XX	B2
**LLS_E91**	Possible male	1Y, 1X	n.d.	B2

**Table 3:** Sequencing statistics.

Sample	Total Reads (nuclear)	Mapped not clonal reads with q ≥ 30	Avg. Read Length	% Human DNA	Avg. coverage mtGenome (rCRS)	avg. mt contamination	Damage at 1st base %	Sex
LLS03	390,156	42,917	51.36	11.00	17	0.57	16	XX
LLS07	300,725	25,223	49.03	8.31	11	0.04	21	XX
LLS21	467,245	18,222	52.78	3.68	13	0.78	12	XY
LLS24	446,180	13,385	61.26	3.01	18	1.46	15	XY
LLS31	395,734	4,749	52.13	1.12	8	0.89	12	not assigned
LLS34	443,334	41,230	56.11	9.31	14	0.36	17	XY
LLS63	395,324	20,161	49.24	5.08	1.2	0.81	19	XY
LLS83	253,204	9099	46.50	0.07	0.4	0.35	11	not assigned
LLS90	415,899	3478	88.27	0.84	0.8	1.2	19	most likely XX

### Variability in cranial modification

**Fig 7:**
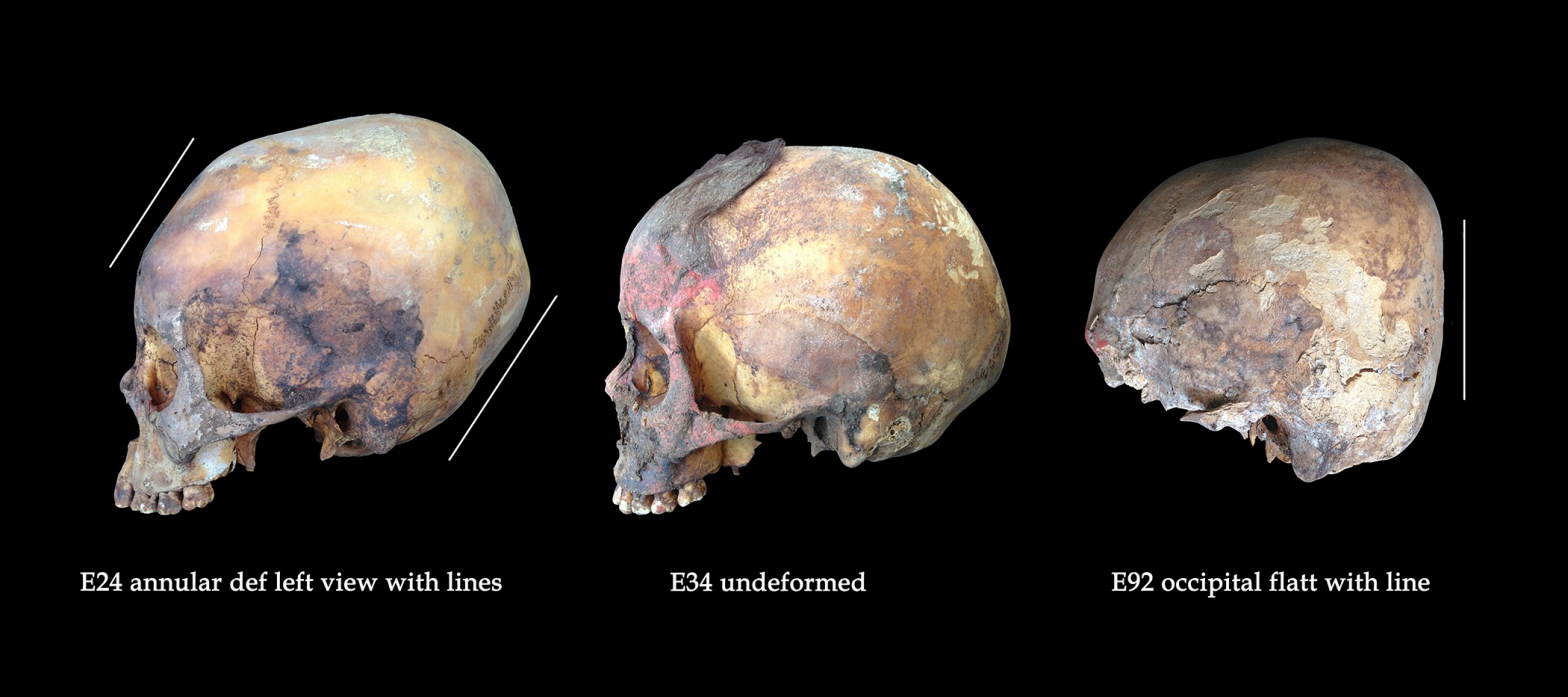
Comparison of variation in cranial shape reflecting distinct forms of cranial modification. On left, annular deformation; at center, unmodified; and at right, occipital flattening.

Variation in styles of cranial modification indicates that the children buried at HLL are a heterogeneous sample, perhaps drawn from distinct ethnic groups and geographic regions (Fig 7). Of 130 crania sufficiently complete enough to be evaluated, 85.4% (111/130) show no cranial modification. Surprisingly, only 8.5% (11/130) show the form of occipital flattening typical of prehistoric populations of the north coast of Peru: tabular erect [66] or fronto-occipital (anteroposterior) deformation [67], which is considered to be the result of cradle boarding in infancy [67–69]. Eight crania (6.2%) show a distinct form of cranial modification known as annular [66] or circumferential [67]. With the exception of a single well-contextualized burial from an early LIP context at the site of El Brujo in the Chicama Valley (who we interpret as a highland woman who migrated to the coast) and seven individuals in a Chimú mass killing in the Huarmey Valley [also interpreted as possibly non-local [21] to our knowledge no other examples of annular deformation have been published from north coast Peruvian sites of any time period. Likewise, fronto-occipital cradleboard deformation has not been reported in the northern highlands, where both unmodified crania and crania with annular deformation have been documented archaeologically [67–69], suggesting that some of the Huanchaquito-Las Llamas children—particularly those with annular deformation—are of highland rather than coastal origin. While annular deformation was practiced in more distant regions, such as the southern highlands and south coast of Peru highland Bolivia and northern Chile, these areas lie far beyond the recognized boundaries of the Chimú state, and we consider these as unlikely places of origin for any of the HLL sacrificial victims.

Unfortunately, there is very little archaeologically excavated human skeletal material from the site of Chan Chan to which the HLL children can be compared. The cemeteries and burial platforms of Chan Chan have been intensively looted since the early Colonial Period (Moseley and Day 1982) and only a limited number of burials have been excavated and studied by biological anthropologists despite extensive fieldwork at the site. To date the largest known sample for which cranial deformation has been recorded is a collection of looted burials surrounding the principal tomb in the burial platform at the Las Avispas compound, excavated by Thomas Pozorski and subsequently re-examined by Andrew Nelson [70]. Nelson’s examination of the Las Avispas crania found that more than half showed fronto-occipital deformation. No examples of annular deformation have been identified at Las Avispas or elsewhere at Chan Chan.

Ongoing excavations at Chan Chan may eventually produce skeletal samples sufficient for comparison with the Huanchaquito-Las Llamas children. For now, we must rely on data from other Late Intermediate Period sites in north coast valleys that were under the control of the Chimú state which can serve as general proxies for deformation practices on the north coast of Peru during the period of Chimú hegemony. Table 4 presents data on cranial deformation frequencies from three Late Intermediate Period sites in coastal valleys to the north of Chan Chan and one from the Huarmey Valley south of Chan Chan. All show a predominance of fronto-occipital deformation, ranging from 63–82%. Compared with these contemporaneous north coast collections the HLL children, with only 8.5% showing fronto-occipital deformation and 6.2% showing annular deformation, stand out as an anomalous sample, supporting the hypothesis that they were drawn from diverse groups and not from a single local population. In contrast, the three sacrificed adults found at HLL (described below) show fronto-occipital deformation typical of northern coastal Peru during the Late Intermediate Period.

**Table 4:** Cranial deformation frequency and form at various north coast LIP sites.

Sample	Number of observable crania	Undeformed n (%)	Fronto-Occipital Deformation n (%)	Annular Deformation n (%)
Pacatnamú Jequetepeque Valley	103	25 (24.3%)	78 (75.7%)	0
El Brujo Chicama Valley	76	9 (11.8%)	48 (63.2%)	1 (1.3%)
Malabrigo Chicama Valley	51	9 (17.6%)	42 (82.4%)	0
Punta Lobos Huarmey Valley	91	16 (17.5%)	68 (74.7%)	7 (7.7%)
Huanchaquito-Las Llamas Children	130	111 (85.4%)	11 (8.5%)	8 (6.2%)
Huanchaquito-Las Llamas Adults	3	0	3 (100%)	0

### Carbon and nitrogen stable isotope analysis

Stable isotope analysis (SIA) in human and animal remains found in archaeological contexts serves as a valuable source of information about their dietary history. Information on stable isotopic analysis of the camelids from Huanchaquito Las Llamas is summarized below and in detail in two recent publications [71]. Preliminary data on the sacrificed children is presented here and compared to data from other northern and central coastal Peru Late Intermediate Period and Late Horizon samples [72, 73].

### Preparation and analysis

Dentin was extracted from 38 HLL tooth samples and purified using the modified Longin method with ultrafiltration following procedures detailed by Brown et al. [74] and Hoggarth et al. [75]. Physically cleaned samples were demineralized and gelatinized. Crude gelatin yields were recorded and then ultrafiltered, retaining > 30 kDa molecular weight gelatin. Carbon and nitrogen concentrations and stable isotope ratios were measured at the Yale Earth Systems Center for Stable Isotopic Studies facility with a Costech elemental analyzer (ECS 4010) and a Thermo DeltaPlus Advantage analyzer. Sample quality was evaluated by percentage crude gelatin yield, C percentage, N percentage, and C:N ratio following van Klinken. [76].

## Results

Stable carbon and nitrogen isotopic ratios from 38 individuals are given in Table 5. In Table 6 summary statistics (mean, range, and standard deviation) are presented and compared with available data from other roughly contemporaneous sites in the Jequetepeque (Pacatnamú), Lambayeque (Chotuna-Chornancap), and Rimac (Puruchuco-Huaquerones) valleys. Very few studies of carbon and nitrogen isotopes are available for northern coastal Peru. For this reason, the Puruchuco-Huaquerones data are included for comparative purposes, even though the Rimac Valley lies just beyond the southern frontier of the Chimú state. As can be seen in Table 6, mean δ^13^C values are quite similar (-11.2 to -11.9) across all samples, but the mean value for δ ^15^N in the HLL sample (8.2) is notably lower. Examination of individual values shows that the most negative δ ^13^C value is found at HLL (-16.0), as is the case for δ ^15^N as well (6.2), both beyond the range of the comparative samples. While the residential and life history of the HLL children is unknown, the variability in their dietary signatures, and some notable outliers supports the inference from cranial modification that they may have been drawn from diverse ethnic groups and/or geographic regions.

**Table 5:** Huanchaquito las llamas isotopic data.

Field #	Age	Tooth	%UF Gelatin	δ ^13^C	δ ^15^N	%C
E01	6.0	dm2	2.7	-12.5	7.1	46.1
E03	12.0	M2	2.9	-14.6	10.3	43.5
E04	12.0	M1	4.4	-12.5	7.6	43.5
E05	15.0	PM	4.7	-10.3	7.7	43.1
E06	9.0	M1	3.6	-12.6	7.9	38.0
E08	10.0	PM	2.9	-11.8	7.2	46.8
E09	11.0	PM	2.1	-12.7	9.7	42.2
E11	12.0	M1	2.4	-9.2	7.6	47.9
E12	11.0	M1	1.7	-10.2	10.9	46.5
E15	12.0	M2	1.4	-9.3	11.5	43.6
E18	15.0	M2	2.6	-13.0	9.0	46.0
E19	11.0	M1	1.8	-13.2	7.5	45.5
E21	6.0	M1	1.6	-10.2	11.7	43.3
E22	7.0	M1	1.9	-10.4	8.2	43.6
E23	9.0	M1	3.9	-10.9	6.9	51.2
E24	10.0	M1	3.3	-13.5	6.7	41.7
E25	15.0	M1	2.2	-9.9	7.2	49.5
E26	10.0	M1	3.8	-16.0	6.3	45.3
E48	10.0	M1	1.5	-12.2	8.8	47.6
E29	10.0	M1	2.6	-13.3	10.4	48.9
E30	9.0	M1	2.8	-11.7	8.5	46.6
E31	9.0	M2	2.1	-9.8	7.7	46.2
E32	7.0	PM	1.4	-10.4	7.2	45.5
E33	8.0	PM	3.5	-12.8	8.3	45.3
E34	8.0	M1	2.6	-13.1	8.2	48.7
E36	9.0	M1	3.6	-11.4	10.4	48.0
E37	7.0	M2	0.1	-9.3	12.2	46.6
E38	12.0	PM	2.5	-10.9	6.7	51.8
E40	9.0	M1	0.9	-12.3	6.2	44.3
E41	7.0	M1	1.3	-10.7	6.6	45.4
E42	8.0	M1	3.4	-15.4	7.6	44.9
E43	7.0	PM	2.7	-13.5	7.7	48.1
E10	10.0	M1	1.1	-12.9	7.8	46.3
E16	10.0	M1	5.6	-14.4	6.5	50.0
E02	13.0	PM	3.4	-13.4	8.3	46.9
E17	6.0	M1	3.7	-10.2	7.4	48.1
E35	12.0	PM	1.5	-10.6	8.4	47.2
E46	7.0	PM	3.2	-12.4	6.5	48.1

**Table 6:** Stable isotopes. Summary statistics for HLL and comparative samples.

Sample	n	Date	Mean δ ^13^C collagen	s.d.	Range	Mean δ ^15^N	s.d.	Range
Puruchuco-Huaquerones^1^	45	Late Horizon	-11.2	1.0	-13.7 to -8.5	10.6	1.2	8.4 to 14.1
Chotuna-Chornancap^2^	25	Late Horizon	-11.8	1.3	-13.9 to -9.1	11.5	1.3	8.9 to 14.0
Pacatnamú^3^	10	Late Intermediate Period	-11.2	1.3	-13.5 to -9.3	11.7	1.6	8.2 to 14.2
Huanchaquito- Las Llamas	38	Late Intermediate Period	-11.9	1.7	-16.0 to -9.2	8.2	1.6	6.2 to 12.2

**Fig 8:**
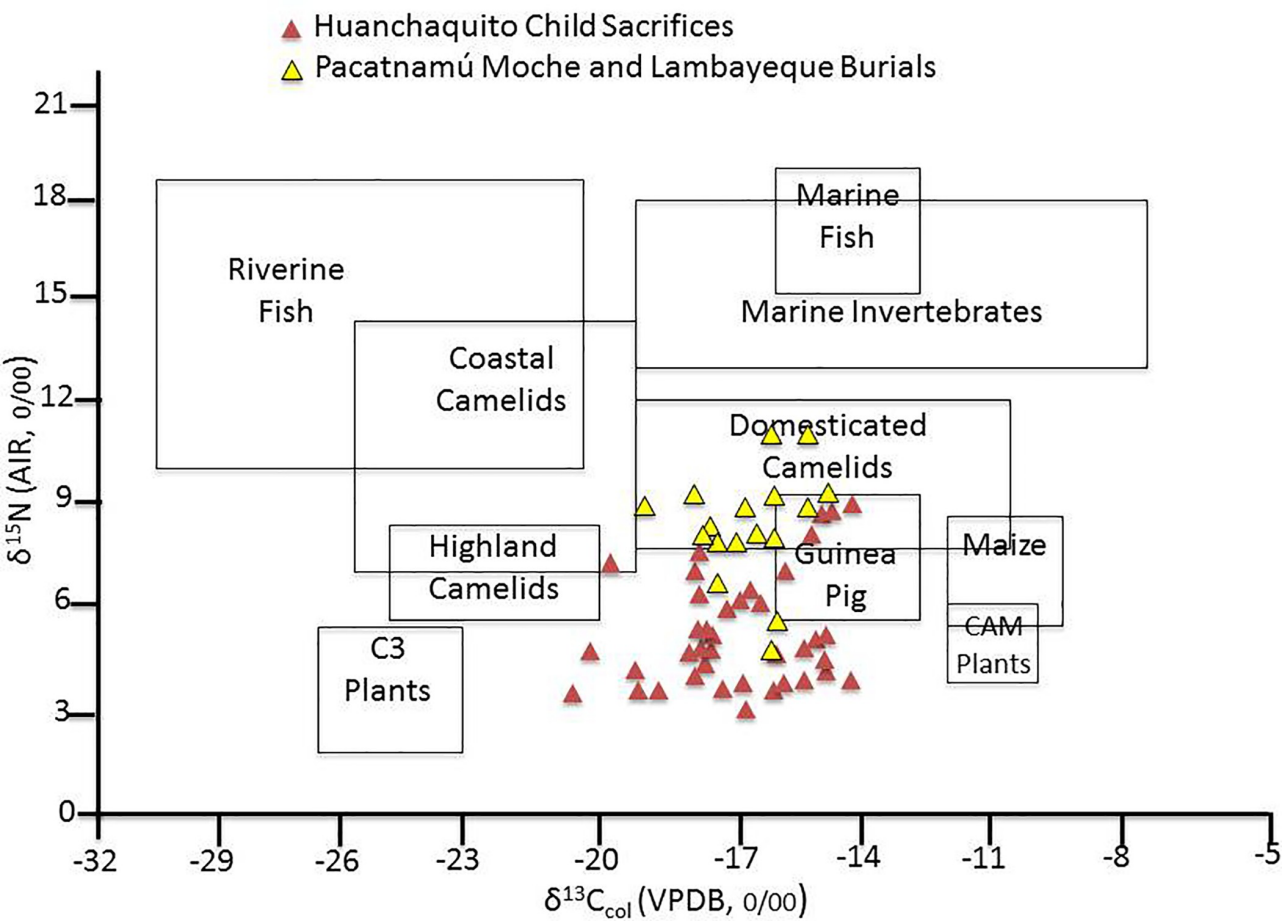
Huanchaquito-Las llamas stable isotopes of carbon and nitrogen (corrected for diet) plotted onto an andean food web, and compared to similar isotopic data from the nearby site of pacatnamú (light triangles).

HLL isotopic data are presented in Fig 8, where collagen values are adjusted to reflect dietary protein [76–77–78–79] and plotted on an Andean food web diagram adapted from Williams and Murphy [57]. For comparison, Fig 8 also includes a plot of similarly adjusted bone collagen values from the geographically and temporally closest comparative sample of LIP burials from the site of Pacatnamú, located on a coastal blufftop in the Jequetepeque Valley, c. 100 km north of Huanchaquito [80]. The HLL children show a wider range of both ^13^C/^12^C and ^15^N/^14^N ratios compared to Pacatnamú, and many have nitrogen values that suggest less marine protein in the diet.

Interpreting the dietary implications of carbon and nitrogen stable isotope values in the HLL sample is complicated by several factors, including (1) potential variability in carbon and nitrogen values in children, based on the developmental timing of specific teeth sampled and weaning age [81], (2) a lack of comparative data from cemetery samples at Chan Chan and other LIP Moche Valley sites, and (3) by evidence of inter-site diversity in diet based on studies of occupational refuse from Chan Chan and surrounding LIP sites conducted by Shelia Pozorski [22]. Despite Chan Chan’s location on a bluff above the Pacific Ocean, Pozorski’s excavations of domestic refuse deposits revealed that the urban population derived the majority of their animal protein not from marine resources but from domestic camelids and to a lesser degree from guinea pigs and dogs, with only a small dietary contribution from fish and shellfish. In contrast, habitation refuse at three satellite communities surrounding Chan Chan (Caracoles, Choroval, and Cerro la Virgen) revealed evidence of more marine protein consumption, although the proportions of terrestrial/marine food sources varied among these sites as well. In general terms, the coastal samples compared in Table 6 show similar carbon and nitrogen isotopic ratios, indicating a diet of mixed C3 and C4 plants (fruits, root crops, beans, and maize) and lower trophic level proteins from largely terrestrial sources such as camelids and guinea pigs [57]. On average, the HLL children appear to have consumed a diet similar to that of other LIP coastal populations, although some individuals have δ ^13^C and δ ^15^N values that depart sharply from the mean values at HLL and at comparative sites. Further investigation of these outliers taking into account tooth type and possible weaning age, as well as by examination of additional stable isotopes and aDNA is currently in progress.

### Osteological evidence of sacrificial method

**Fig 9:**
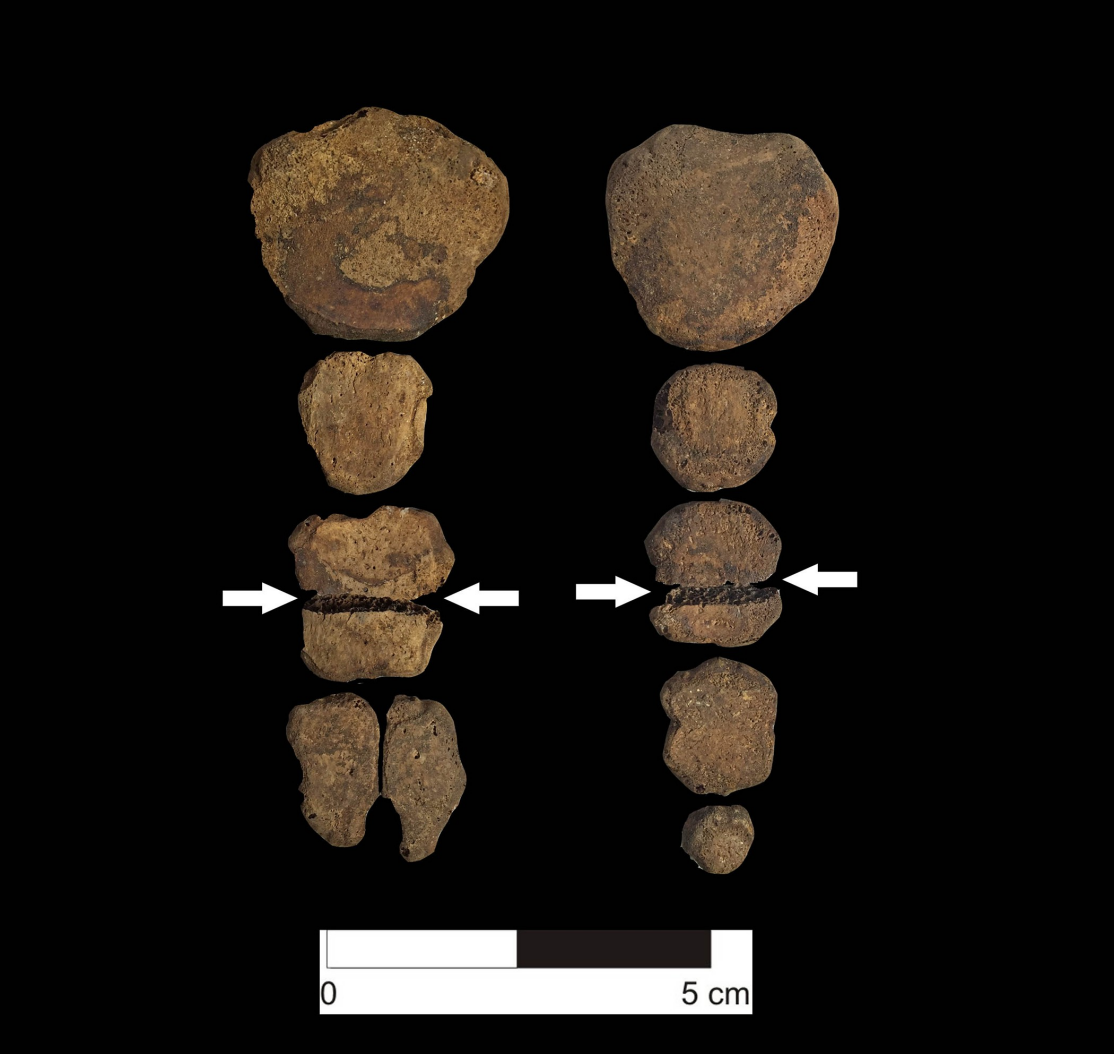
Examples of bisected sternal elements.

Laboratory examination revealed that nearly all children with complete sternal elements showed a single transverse cut through one of the sternebrae (unfused sternal elements). The cuts are consistent in location, angle and direction, and the rarity of hesitation cuts or “false starts” [82] suggests that an experienced hand made them (Fig 9). Approximately 10% also showed cut marks on the external surface of the third or fourth rib, as was seen in the camelids as well (see below). Many of children had visible spreading and displacement of the ribs, indicating that the chest was opened forcefully.

The transverse orientation of the cuts of the sterna of the Huanchaquito children are unlike those seen in any other sacrificial victims from ancient Peru. While cutting open of the chest of sacrificial victims by an oblique cut through the manubrium sterni has been documented at the north coast Peruvian sites of Pacatnamú [83], Túcume [16–17] Cerro Cerrillos, and Chotuna-Chornancap [14, 84], the approach to the thoracic cavity at Huanchaquito is different. Accessing the heart by transverse sectioning of the sternum is a technique familiar to modern thoracic surgeons, and is known by various names (e.g., bilateral anterior thoracotomy with transverse sternotomy, transsternal bilateral thoracotomy, and “clamshell” incision [57, 85–86–87]. The purpose of opening the chests of the children (and camelids; see below) at HLL can only be hypothesized, but heart removal is a likely motivation. Soft tissue preservation was not sufficient to demonstrate that the Huanchaquito children’s hearts were removed, but some ethnohistoric accounts of ritual practices of the Inca describe heart removal as a sacrificial method. For example, the sixteenth century Spanish chronicler Cristóbal de Molina described various forms of Inca child sacrifice, including extraction of the heart. In his *Account of the Fables and Rites of the Incas*, he wrote “Other [children] had their live hearts taken out, and so the priests offered the beating [hearts] to the huacas to which the sacrifice was made.” [88]. While the veracity of Molina’s account cannot be confirmed, extraction of the hearts of camelids was a form of sacrifice also described and illustrated by chroniclers in the sixteenth and seventeenth century, and it is still practiced in modern highland Peru (see below).

### Zooarchaeological study of camelid remains

More than 200 camelids were excavated at HLL. Preliminary analysis has been conducted on 120 specimens. Although not all camelid remains were complete due to post-depositional disturbance, the overall preservation was excellent and allowed the study of perishable materials such as wool, stomach contents, sedge ropes, and plant remains caught in the animals’ hair, observations that are not normally observable in archaeological contexts. It is very difficult to differentiate llama (*Lama glama*) from alpaca (*Lama pacos*) from an osteological point of view. Moreover, the osteometrical approach based on the first phalanx first proposed by Kent [89] and revised by Izeta [90] could not be used due to the young age of the camelids. We thus used dental morphology (incisor shape and location of enamel) and wool characteristics (fiber morphology and pattern, distribution of colors) that indicate that the camelids were probably llamas (*Lama glama*).

Age was estimated on the basis of dental eruption and tooth wear [91]. All the camelids were immature, less than a year and a half old, with 75% estimated to be less than 9 months of age. The very high proportion of very young individuals and the lack of adults indicate that these animals were age-selected. There is a clear parallel between the young ages of the children and the camelids.

A variety of coat colors was observed, including beige, light brown, dark brown and mixed colors such as a brown background with beige dots. The most frequently observed color was brown; the least common was beige. The predominance of brown and mixed color, along with the young age of the animals, appear to have been principal criteria in the selection of animals for sacrifice. Some colors may have been considered most appropriate for sacrificial rituals. Although the possible symbolic significance of color selection is not known for Chimú society, comparative data are available for the Inca. Spanish chroniclers described in detail the ritual sacrifices of camelids according to the Inca ceremonial calendar, and noted that specific colors of camelids were selected for particular events and seasons [92].

**Fig 10:**
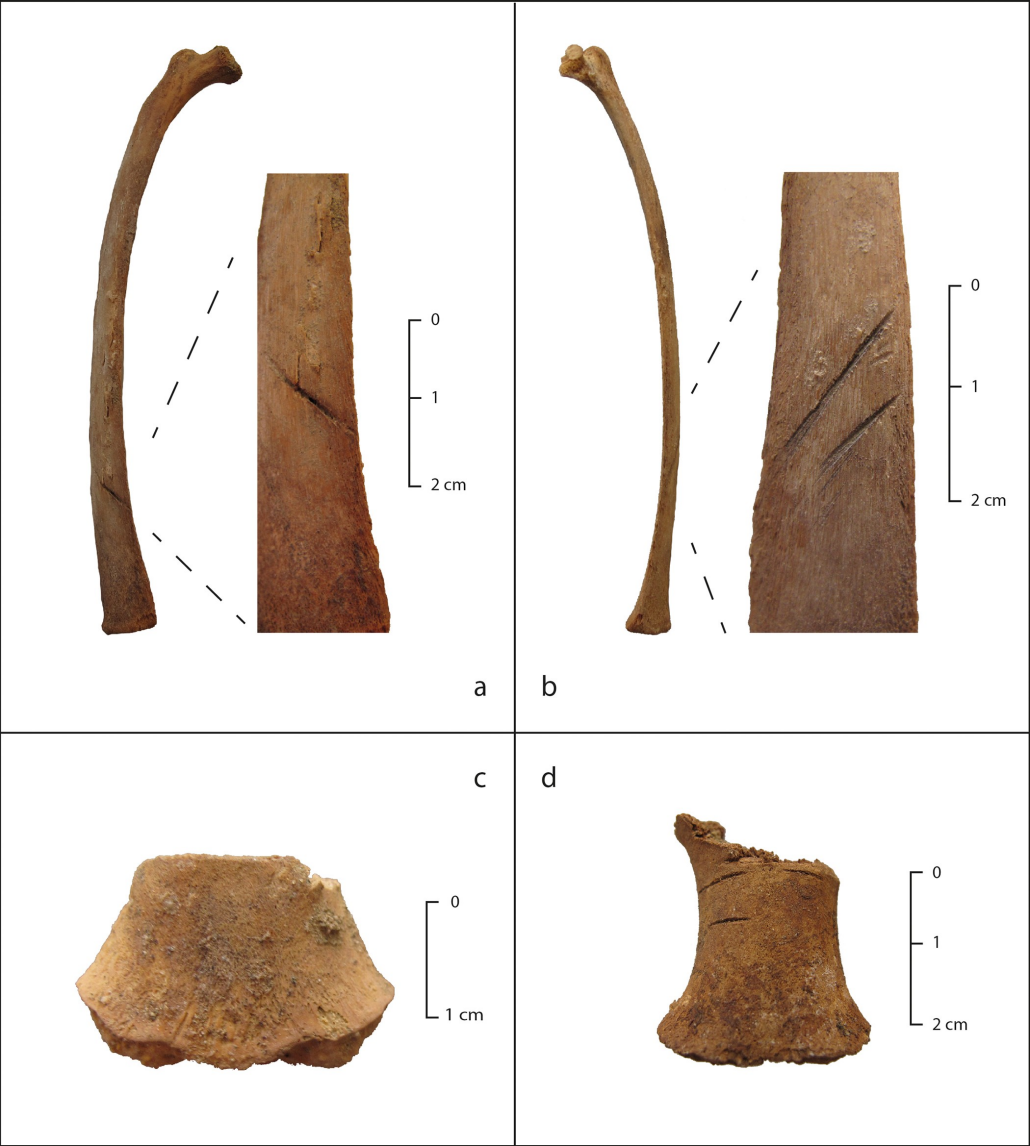
Cut marks on ribs and sternebrae of camelids.

At HLL particular attention was paid to determining how the camelids were sacrificed. No evidence was found of blunt force trauma to the skull or slashing of the throat. Like the children, the camelids show transverse cuts through the sternebrae, predominantly the second and third (Fig 10). Cut marks are often present on the ribs as well, most frequently on the external shaft of the third and fourth ribs (both right and left). Miller [93–94] recorded cut marks on camelid sterna and ribs made by a sacrificial technique known in the Peruvian highlands as the *ch’illa*, which is still practiced today. This technique is used to remove the heart after inserting the hand and forearm into the chest of the animal. Although the location of cut marks on HLL camelids differs somewhat from that described by Miller in his ethnographic study, there are clear parallels between the two. Perhaps the young age of the camelids, with more fragile bones of those of an adult, did not require the same location of chest opening.

**Fig 11:**
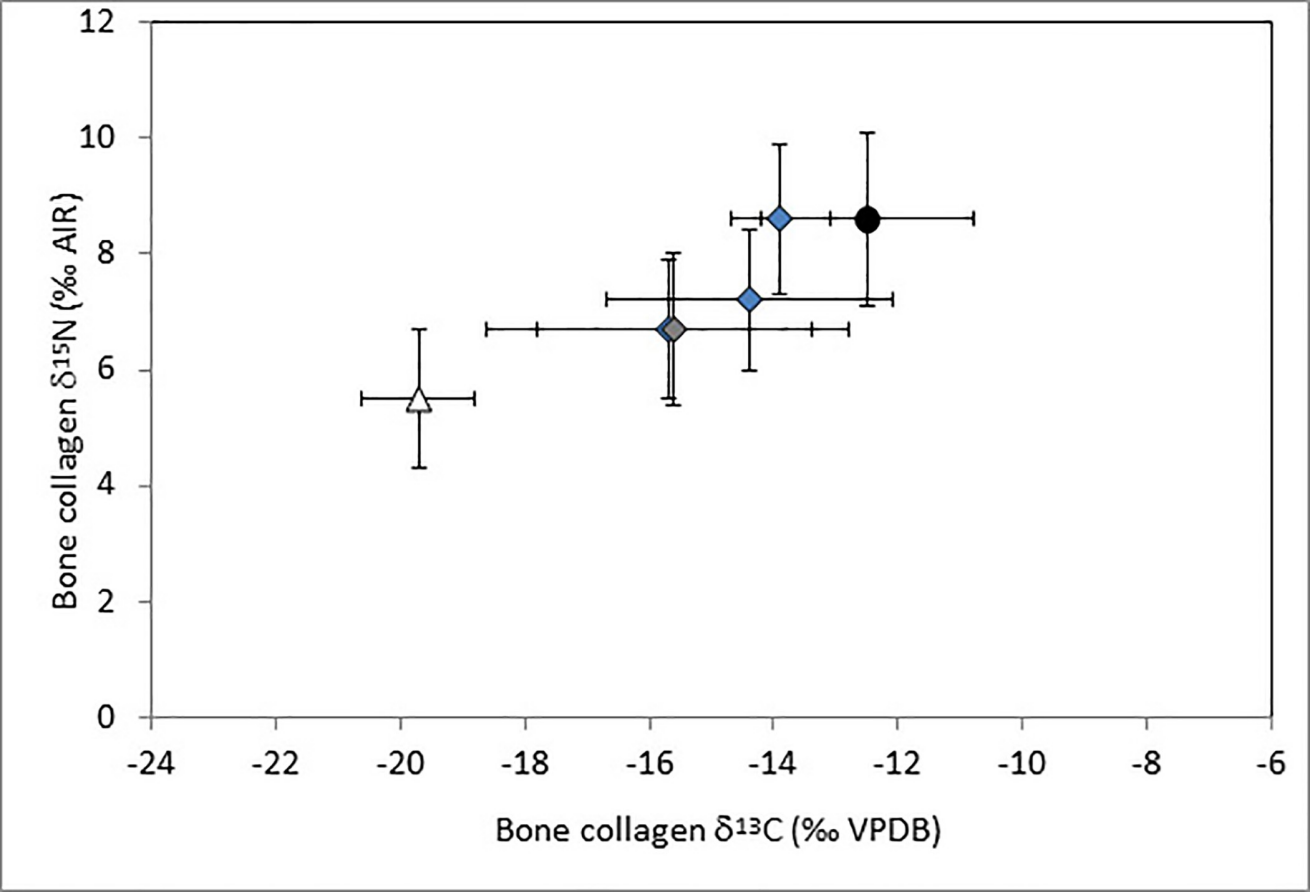
Comparison of bone collagen δ^13^C (‰ VPDB) and δ^15^N (‰ AIR) mean values (± 1SD) of sacrificed camelids from huanchaquito-las lamas (dark circle) compared with modern specimens (open triangle; Dufour *et al*. 2104) from north peruvian highlands, archaeological specimens from the LIP to MH site of huaca santa clara (grey diamond; szpak *et al*. 2014) and early intermediate period sites of el brujo, huaca santa clara and huaca gallinazo (blue diamonds; dufour *et al*. 2014; szpak *et al*. 2014). Modern specimen values are reported with a +1.5 ‰ adjustment factor to account for modern enrichment of atmosphere in ^12^C (Suess Effect).

In order to investigate the geographical origin of the sacrificed camelids, a preliminary isotopic analysis was conducted on 29 animals. Bone collagen was extracted following the protocol of Bocherens et al. [95]. Following demineralization of powdered bone, potential contamination of fulvic and humic acids were removed. Gelatin was combusted using an Elemental Analyser Flash 2000, coupled with a Delta V Advantage (Thermo Scientific) isotope ratio mass spectrometer for δ^13^C and δ^15^N analysis at the Service de Spectrométrie de Masse Isotopique du Muséum national d’Histoire naturelle (MNHN, Paris, France). Extraction yield (mean = 15.1 ± 4.2%) and C:N ratio (mean 3.2 ± 0.1) indicate the a very good quality of preservation of HLL specimens. Stable isotopic analysis of collagen extracted from bone has been demonstrated to be an appropriate method for distinguishing between individuals raised at high and low altitudes in the Andes [96–97]. The western slopes of the Andes present a wide variety of ecological zones [98] from the coast to the highlands, characterized by differences in physical parameters such as aridity and availability of different food resource categories such as C_3_ *versus* C_4_ plants [99]. δ^13^C values of all individuals tested indicate the consumption of C_4_ plants–a photosynthetic pathway largely absent in the elevated areas of the Andes (Fig 11). δ^15^N values further suggest that camelids were raised in an arid environment typical of the coast and mid-valley. These values are consistent with previously reported data for Early Intermediate Period contexts [96–97] and confirm local herding practices specific to pre-Hispanic times in northern coastal Peru.

## Conclusions

The archaeological context and osteological analysis of the human and camelid remains at Huanchaquito document a mass sacrifice of children and camelids on a scale unlike any seen previously in Andean South America. In number, it greatly exceeds the known sample of Inca child sacrifices from high altitude sites in the Andes [3, 38, 40]. It also is substantially larger than the only other mass sacrifice of children known from the New World, that of 42 children in Offering 48 at the Mexica Templo Mayor in Central Mexico [36]. Relatively few convincing examples of child sacrifice are known from the Old World [100], and in most cases, there is debate over whether these in fact can be identified as intentional killing, given a lack of osteological evidence of cause of death [30, 101]. In the case of HLL, there is no such ambiguity. Skeletal evidence clearly indicates that the children and camelids were sacrificed by cutting open the thoracic cavity. No other evidence of perimortem (occurring at or around the time of death) trauma was observed in any of the children or camelids, indicating that the sacrificial program was a consistent one. Although human sacrifice by opening the thoracic cavity has been documented previously at north coast Peruvian sites, the specific technique used at Huanchaquito is unlike that of previously documented cases in Peru. Three adult skeletons found in direct association with the children and camelids also appear to be sacrifices, based on their atypical burial positions (crouched face down or splayed on the back), evidence of blunt force trauma in the two females, and a lack of funerary offerings, as well as their close physical association with the child and camelid burials, although their chests were not opened.

Variation in forms of cranial deformation and the wide range of carbon and nitrogen isotopic ratios observed in the children suggest that they are a heterogeneous sample, perhaps composed of individuals selected from various geographic or ethnic groups, rather than from a single local population, similar to what has been found in Inca child sacrifices [4, 38, 42–43]. The geographic origins and life histories of the HLL children, who range from approximately five to fourteen years of age, is a question to be explored further through ongoing laboratory analyses, including the study of additional stable isotopes (to examine diet and geographic origin), aDNA (to determine sex and possible genetic relationships with other coastal and highland groups), and through analysis of skeletal indicators of health and cultural markers such as intentional cranial modification. The presence of a thick layer of mud on top of the sand in which the children and camelids were buried, as well as the presence of human and animal footprints made while the mud was still wet, suggest that the sacrificial event occurred shortly after heavy rainfall and flooding, in an arid region that receives negligible rainfall under normal conditions. While the correlation between heavy rains and the sacrifice may be coincidental, it is tempting to hypothesize that the two events are associated, and that the mass offering of children and camelids may have been an attempt to appease the gods and mitigate the effects of a major ENSO event that occurred around 1400–1450 A.D. The sacrifice of such a large number of children and camelids constituted a significant investment of resources for the Chimú state, whose massive capital city Chan Chan lies less than a kilometer away from the Huanchaquito Las Llamas sacrifice site.
